# Exploring the cell–protein–mineral interfaces: Interplay of silica (nano)rods@collagen biocomposites with human dermal fibroblasts

**DOI:** 10.1016/j.mtbio.2019.100004

**Published:** 2019-04-08

**Authors:** Yupeng Shi, Christophe Hélary, Thibaud Coradin

**Affiliations:** Sorbonne Université, CNRS, Collège de France, Laboratoire de Chimie de la Matière Condensée de Paris, 4 Place Jussieu, 75005 Paris, France

**Keywords:** Nanocomposites, Collagen, Silica, Biointerfaces

## Abstract

The benefits of associating biological polymers with nanomaterials within functional bionanocomposite hydrogels have already been evidenced both *in vitro* and *in vivo*. However their development as effective biomaterials requires to understand and tune the interactions at the cell–protein–mineral ternary interface. With this purpose, we have studied here the impact of silica (nano)rods on the structural and rheological properties of type I collagen hydrogels ​and on the behavior of human dermal fibroblasts. High collagen concentrations were beneficial to the material mechanical properties, whereas silica rods could exert a positive effect on these at both low and high content. Electron microscopy evidenced strong bio–mineral interactions, emphasizing the true composite nature of these materials. In contrast, adhesion and proliferation studies showed that, despite these interactions, fibroblasts can discriminate between the protein and the inorganic phases and penetrate the collagen network to limit direct contact with silica. Such a divergence between physicochemical characteristics and biological responses has major implications for the prediction of the *in vivo* fate of nanocomposite biomaterials.

## Highlights

• Bionanocomposites were prepared from collagen hydrogels and silica nanorods.
• Rheological properties reflect the composite nature of the materials.
• Strong bio–mineral interactions are evidenced by silica–collagen coalignment.
• Human fibroblasts discriminate between the protein and the mineral phases.

## Graphical abstract

**Figure:**
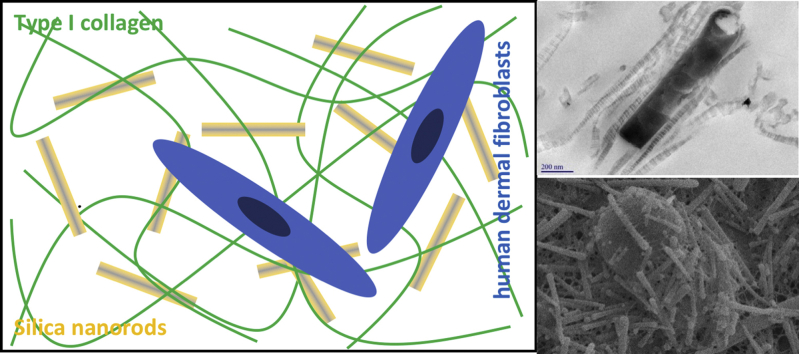

## 1 Introduction

Hydrogels prepared from natural biomolecules have a broad field of applications, including tissue engineering, drug delivery, and soft electronics [1], [2], [3], [4]. However, except when prepared at very high density[5], they generally have limited intrinsic mechanical properties and fast degradation rates [6]. As an alternative to chemical or physical cross-linking[7], incorporation of nanoscale fillers into biohydrogels to form bionanocomposites has recently emerged as a versatile and efficient approach, not only to address these issues but also to create materials with new functions[8], [9], [10], [11].

When designing particle–matrix composites, optimal enhancement of the elastic properties can be obtained if the added charges form a percolated network [12]. Because the percolation threshold decreases with increasing aspect ratio of the fillers, anisotropic particles are usually preferred [13]. The adhesion strength between the matrix and the particle surface is also of major importance [14] and can be tuned by modifying the chemical nature of the charge. However, when designing nanocomposite biomaterials, additional constraints must be taken into account. Indeed the charges must be non-toxic, but it is also important to consider the affinity of cells for the particle surface they will sense when exploring their environment. Such an affinity depends not only on the surface chemistry but also on the particle dimension and shape [15], [16]. The topology of the composite network depending on the density and dispersion state of the charges will also impact cell behavior [17]. Whereas these principles are well studied in 2D configurations, their extension to 3D systems where cells have access to an additional dimension for interaction and mobility remain scarcely studied [18], [19].

A particularly interesting family of hydrogels to further investigate these questions is that based on type I collagen, whose strong affinity for many mammalians cells and excellent biocompatibility make it a protein of choice to prepare biomaterials [5], [20], [21]. Collagen-based hydrogels are already widely used for bone, cartilage, and skin repair [22], [23], [24]. In the field of bionanocomposites, the most studied approach relies on the introduction of hydroxyapatite nanoparticles following a bone-mimicking strategy [25], [26], [27]. Other nanoparticles associated with type I collagen include carbon nanotubes to produce fiber-reinforced composites and prepare electrically conductive hydrogels [28], [29], metallic colloids for tissue engineering applications [30], magnetic iron oxide for guided nerve repair [31], and, very recently, ZnO nanoparticles [32].

Silica nanoparticles were also widely used to design collagen-based nanocomposites [33]. Desimone et al. [34], [35] prepared silica nanoparticle–collagen composite scaffolds for fibroblast cells encapsulation, and then evidenced their biocompatibility. Further developments towards 3D matrices for bone repair, especially using bioglass nanoparticles [36], as well as for the elaboration of medicated wound dressings [37], [38], were reported. The fact that only spherical silica nanoparticles have been so far used explain why reported improvement in mechanical properties remain modest [34], [37], [39]. Gathered evidences indicate that collagen can interact with silica nanosurfaces via attractive electrostatic interactions [40], but the influence of particle morphology on its interaction with collagen has never been reported.

The interactions of mammalian cells with silica nanoparticles in solution has been very extensively studied to elucidate the effect of both intrinsic (including size, morphology, surface chemistry, and internal structure) and external (type of cells, culture medium, concentration, and contact time) parameters [41], [42]. Information available about cell behavior on nanostructured silica surfaces points out the key role of adhesion protein–silica interactions, that highly depend on surface topology and chemistry, [43], [44]. In the case of collagen–silica nanocomposites, both particle size and concentration were shown impact on fibroblast adhesion in 2D and proliferation in 2D and 3D [34], [39]. A combination of factors including modification of the surface chemistry and rugosity, as well as change in the mechanical properties, has been proposed to explain these differences.

In this context, we hypothesized that the use of highly anisotropic silica particles with one dimension comparable to the scale of collagen fibrils and fibroblast cells would allow to promote, and therefore better study, interactions between the different components of the ternary protein–mineral–cell system. To achieve this goal, we used silica nanorods (SiNRs) and prepared new collagen-based composite hydrogels within a wide range of concentrations and ratios. Their structural and rheological properties, as well as their influence on human dermal fibroblast adhesion and proliferation, were studied as a function of protein and nanoparticle content. Mechanical responses were correlated to different regimes of bio–mineral interactions, whereas the fate of seeded cells could be linked to the intrinsic properties of each component of the nanocomposite hydrogel.

## 2 Materials and methods

### 2.1 Synthesis of SiNRs

The rod-like silica particles were synthesized according to the protocol reported by Kuijk et al. [45] Typically, 30 ​g of polyvinylpyrrolidone (PVP, 40 ​kDa) was added to 300 ​mL of *n*-pentanol and sonicated for 3 ​h. Then 30 ​mL of absolute ethanol, 8.4 ​mL of ultrapure water, and 2 ​mL of a 0.18 ​M sodium citrate dihydrate aqueous solution were added to the solution under mild stirring. Then, 6.75 ​mL of ammonia and 3 ​mL of tetraethylorthosilicate were added, and the mixture was shaken manually for a few minutes before placing the glassware into a water bath at 30 ​°C for 24 ​h. Next, the reaction mixture was centrifuged at 2000 g for 20 ​min. The supernatant was removed, and the particles were redispersed in ethanol. This centrifugation procedure was repeated at 1000 ​g for 15 ​min, 2 times with ethanol, 2 times with water, and finally again with ethanol. To improve size dispersity, the rods were centrifuged two times at 500 ​g for 15 ​min and redispersed in fresh ethanol.

### 2.2 Preparation of collagen-based nanocomposites

Type I collagen was purified from rat tails, and the final concentration was estimated by hydroxyproline titration [46]. Nanocomposites were prepared by mixing 5, 10, 20, and 30 ​mg ​mL^−1^ of collagen solutions in 17 ​mM of acetic acid solution with a 10 × phosphate buffer saline (PBS) solution containing the suitable amount of silica rods to reach a final pH of 7.0 and a final concentration of silica rods ranging from 30 to 120 ​mg ​mL^−1^. Resulting sols were quickly dispatched into shaped molds and incubated at 37 ​°C to trigger gel formation. Finally, gels were rinsed three times with PBS.

### 2.3 Electron microscopy analysis

Before scanning electron microscopy (SEM) analysis, collagen nanocomposites were fixed in dimethyl citrate/sucrose buffer (0.05 ​M/0.3 ​M, pH 7.4) at 4 ​°C for 1 ​h using 3.63% glutaraldehyde. Samples were washed three times in the same buffer and dehydrated in water/ethanol baths of increasing alcohol concentration. They were freeze-dried and sputtered with gold (20 ​nm) for analysis. Samples were observed with a Hitachi S-3400 ​N SEM operating at 8 ​kV or 10 ​kV. For transmission electron microscopy (TEM) analysis, the collagen nanocomposites were fixed with 4% Performic acid (PFA) in PBS. After washing, samples were fixed using 2% osmium tetroxide in cacodylate/sucrose buffer (0.05 ​M/0.3 ​M, pH 7.4) at 4 ​°C for 1 ​h. After three washings in cacodylate/sucrose buffer, they were dehydrated with ethanol and embedded in araldite. Thin araldite transverse sections (100–200 ​nm) were performed by Ultracut ultramicrotome (Reichert, France) and stained with phosphotungstic acid. They were imaged using a Tecnai spirit G2 electron microscope operating at 120 ​kV.

### 2.4 Rheological measurements

Shear oscillation measurements were performed on collagen nanocomposite discs using a Bohlin Gemini rheometer (Malvern) equipped with a flat acrylic 40-mm diameter geometry. All tests were performed at 37 ​°C. The mechanical spectra were obtained using a 1% applied strain. In order to test all collagen matrices under similar conditions, the gap between the base and the geometry was chosen before each run so that a slight positive normal force was exerted on the gel during the measurement. Four samples of each nanocomposite hydrogel were tested (*n* ​= ​4).

### 2.5 Cellular studies

Normal human dermal fibroblasts (NHDFs) were grown in complete cell culture medium (Dulbecco's Modified Eagle's Medium with GlutaMAX™, without phenol red supplement, with 10% fetal serum, 100 U ​mL^−1^ penicillin, 100 ​μg/mL streptomycin). Tissue culture flasks (75 ​cm^2^) were kept at 37 ​°C in a 95% air: 5% CO_2_ atmosphere. Before confluence, the cells were removed from flasks by treatment with 0.1% trypsin and 0.02% EDTA, rinsed and resuspended in the culture medium.

For viability test, NHDFs were seeded onto the hydrogel discs at a density of 5000 ​cells/cm^2^. Following 24 ​h of culture, cell activity was evaluated by the Alamar Blue assay. Control experiments were performed using the pure collagen hydrogel. All experiments were performed as tetraplicates (*n* ​= ​4). For adhesion and proliferation assessments, NHDFs were seeded onto the hydrogel discs prepared in 24-well plates at a density of 5000 ​cells/cm^2^. Following 24 ​h, 48 ​h, and 72 ​h of culture, cells on hydrogel surfaces were washed twice with PBS and treated with a 4% PFA solution in PBS for 1 ​h. Cells were then stained with 4,6-diamidino-2-phenylindole (DAPI) and imaged with a LEICA microscope. Fluorescence images were transformed into 8-bit digital grayscale images using ImageJ software, followed by the selection of a threshold on gray levels in order to separate lighter appearing cells from the darker background.

### 2.6 Statistical analysis

Graphical results are presented as mean ​± ​SD (standard deviation). Statistical significance was assessed using one-way analysis of variance (ANOVA) followed by Tukey (compare all pairs of groups) or Dunnett (compare a control group with other groups) post hoc test. The level of significance in all statistical analyses was set at a probability of P ​< ​0.05.

## 3 Results

### 3.1 SiNR ​characterization

**Fig. 1:**
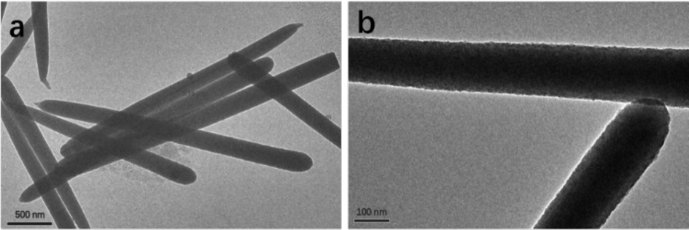
TEM images of silica nanorods. Scale bar (a) 500 nm, (b) 100 nm. TEM, transmission electron microscopy.

As shown on TEM images in Fig. 1, the synthesized silica (nano)rods (SiNR) exhibit good homogeneity, no spherical structures appear, and the rods have a diameter of about 200 ​nm and a length of 3 ​μm (*i.e.* the aspect ratio is 1:15). More precisely, statistical analysis performed over 50 particles indicates dimensions of 245 ​± ​64 ​nm over 3.3 ​± ​0.8 ​μm. Their nail-like morphology, with one end being rather flat and the other end being like a tip, is reminiscent of their growth mechanism [45]. Attempts to obtain reliable size distribution using dynamic light scattering were unsuccessful because of the micron-size length of the rods. It was nevertheless possible to obtain a stable measurement of the zeta potential of prepared SiNRs at −53.8 ​eV, similar to the surface charge of similar silica nanomaterials [47]. Fourier transform infrared spectroscopy confirmed the chemical nature of the rods and showed a minor presence of PVP (Supplementary Fig. S1).

### 3.2 Structural properties of SiNRs–collagen composites

**Fig. 2:**
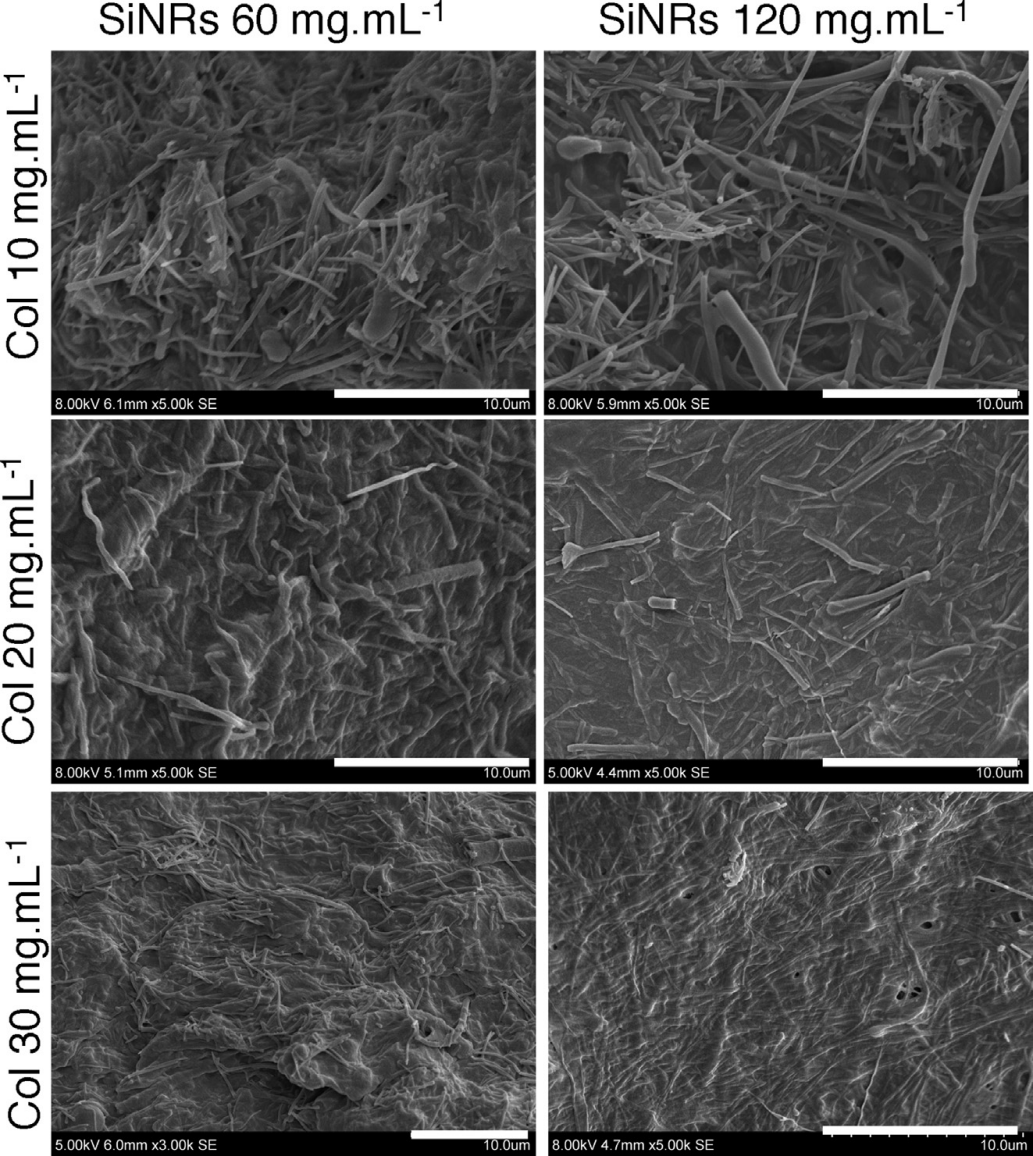
Selected SEM images of SiNRs–collagen nanocomposites at collagen concentrations 10, 20, and 30 ​mg ​mL^−1^ and silica concentration of 60 and 120 ​mg ​mL^−1^ (scale bar ​= ​10 ​μm). SEM, scanning electron microscopy; SiNRs, silica nanorods.

The prepared silica rods were mixed with the collagen solution in acidic conditions under gentle stirring, followed by adjusting the pH value 7 with PBS to yield the SiNRs–collagen hydrogel nanocomposites. Nanocomposite materials were prepared with collagen concentrations of 10 ​mg ​mL^−1^, 20 ​mg ​mL^−1^, and 30 ​mg ​mL^−1^ and silica rod final concentrations from 30 ​mg ​mL^−1^ to 120 ​mg ​mL^−1^. Selected SEM images have been gathered in Fig. 2, and the whole set of images is available as Supplementary Fig. S2.

At 10 ​mg ​mL^−1^ collagen, rods are initially difficult to identify within the highly porous fibrillar network. They become more and more visible with higher SiNR amounts and at 120 ​mg ​mL^−1^ silica, particles resembling rods coated with some organic material are observed. At 20 ​mg ​mL^−1^ collagen, the material appears denser, and rods are observed either on the surface or buried inside the protein network. Almost the same trend is observed at 30 ​mg ​mL^−1^. Noticeably, hydrogel morphology as observed by SEM can strongly vary with drying conditions, and surface (‘crust’) effects are often detrimental to their accurate study by this technique.

**Fig. 3:**
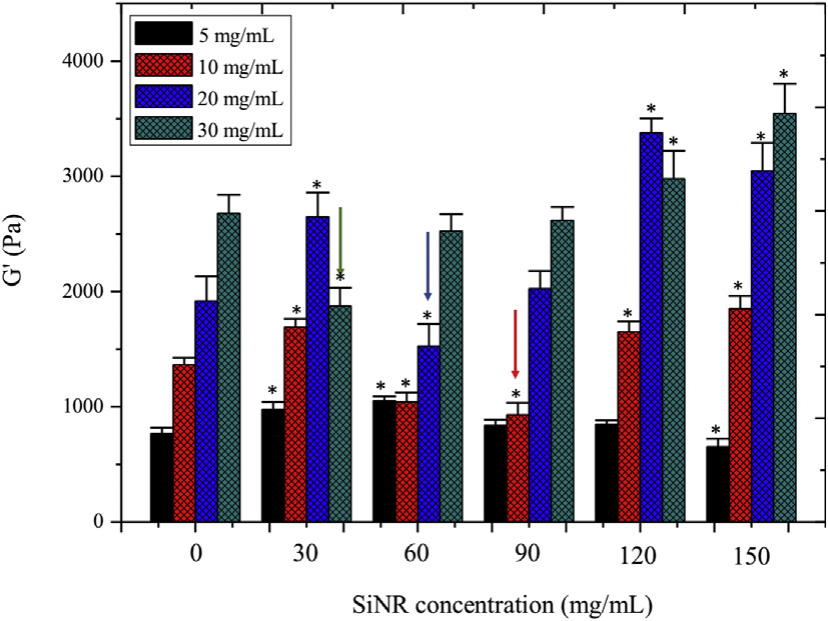
Storage modulus (G′) of SiNR–collagen composite hydrogels with various collagen and silica concentrations. Variance of the G′ value between the hydrogels with same collagen concentration was determined by one-way ANOVA with Dunnett post hoc test, ^∗^P ​< ​0.05. Arrows indicate the SiNR concentration for minimal G′ value. SiNR, silica nanorod; ANOVA, analysis of variance.

The rheological properties of the nanocomposites were investigated and compared to pure collagen hydrogels (Fig. 3 and Supplementary Fig S3). For all materials, the storage modulus *G’* was much higher than the loss modulus *G”*, as expected for hydrogels with elastic properties. The measured moduli for the nanocomposites vary from *ca*. 750–3500 ​Pa.

As a general trend, at a given silica content, *G’* increased with collagen concentration. At a fixed collagen concentration, the evolution of *G’* with SiNR content consisted of an initial small increase followed by a marked decrease and then a new increase for the highest silica concentration. However, the higher the collagen concentration, the lower the SiNRs concentration for which G’ is minimal (see arrows on Fig. 3).

**Fig. 4:**
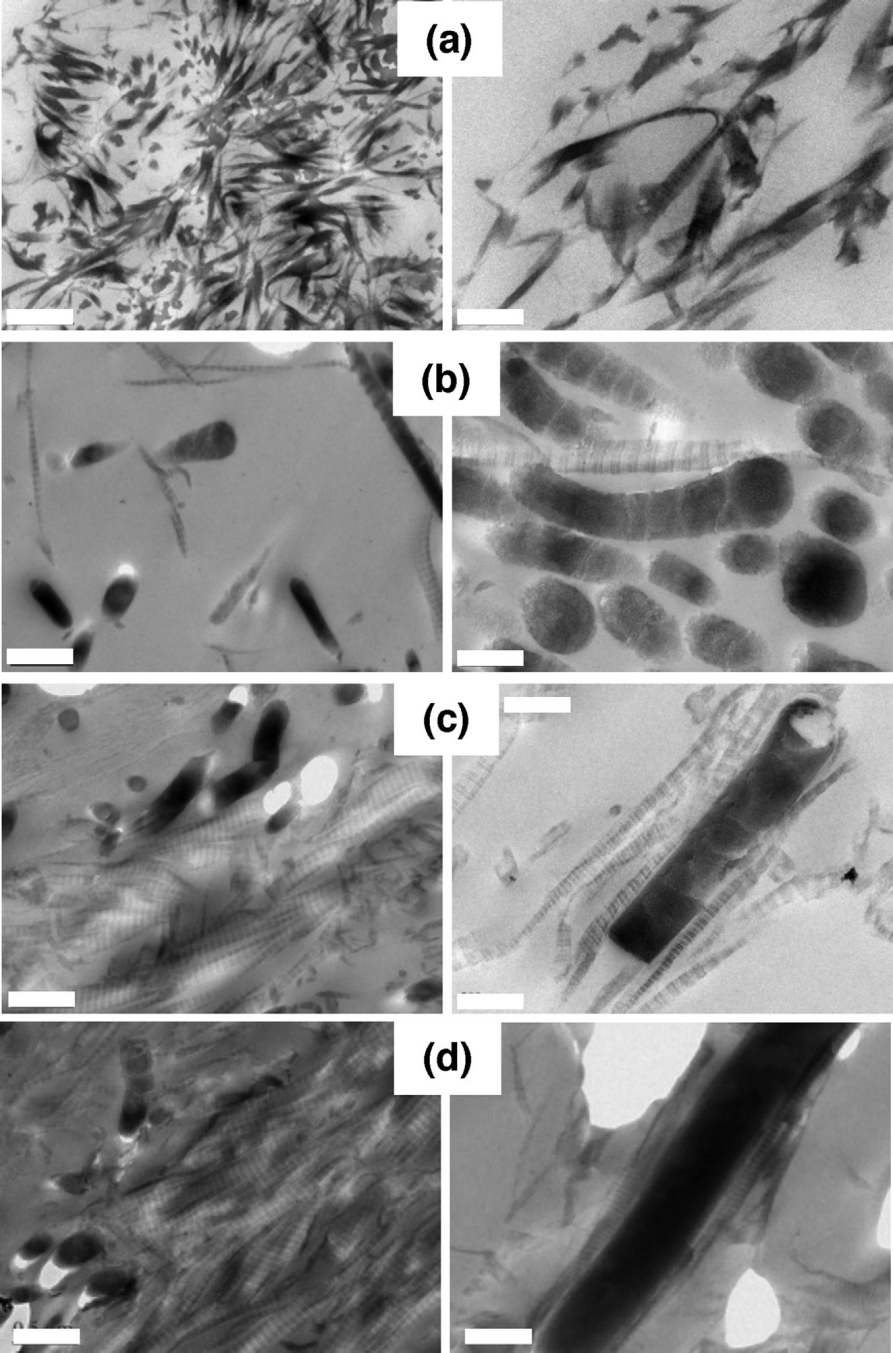
TEM of (a) 20 ​mg ​mL^−1^ collagen. (b) 10 ​mg ​mL^−1^ collagen, 120 ​mg ​mL^−1^ SiNRs. (c) 20 ​mg ​mL^−1^ collagen, 60 ​mg ​mL^−1^ SiNRs. (d) 30 ​mg ​mL^−1^ collagen, 30 ​mg ​mL^−1^ SiNRs. Scale bar: left-hand column: 500 ​nm; right-hand column: 200 ​nm. SiNRs, silica nanorods; TEM, transmission electron microscopy.

To understand better the relationship between nanocomposite structure and mechanical response, we selected hydrogels with similar rheological behaviors but different collagen/SiNR concentrations that were further imaged by TEM (Fig. 4).

The pure collagen hydrogel with a concentration of 20 ​mg ​mL^−1^ showed an obvious fibrous collagen network with densely packed fibrils exhibiting a periodic band pattern of 67 ​nm, which is a typical feature of the physiological structure of type I collagen (Fig. 4a). At 10 ​mg ​mL^−1^ collagen and 120 ​mg ​mL^−1^ silica (Fig. 4b), it was possible to visualize both striated collagen fibrils and other objects that we attributed to sections of rods cut at different orientations. This attribution was supported at higher magnification where various sections of the rods were clearly observed. Interestingly, when longitudinal sections are present, it is possible to observe that, during the sample preparation, the ultramicrotome knife has fragmented the rods in slices, that could reflect the layer-by-layer growth mechanism of such rods [45]. The rods appear highly densely packed, in agreement with the high silica concentration, and at least one large striated collagen fiber can be found in between the elongated mineral particles.

When increasing collagen concentration and decreasing SiNR concentration (Fig. 4c), many collagen fibrils are seen and less rod sections. At higher magnification, a very interesting phenomenon appears: several striated fibers are observed that are aligned along the direction of the rods. Such an alignment not only concerns those fibers that are in contact with the silica surface but at least two other layers of collagen fibers also follow this direction. Finally, increasing further the collagen:silica weight ratio to 1:1 (Fig. 4d), a very dense network of collagen is observed in which some rods appear to be buried. At higher magnification, the silica rods appear surrounded by a continuous sheath of dense and well-aligned collagen fibers.

### 3.4 Interactions of fibroblasts with SiNR–collagen nanocomposites

It is now well admitted that the behavior of cells is highly dependent on the substrate mechanical properties [48]. Here, we had the possibility to prepare materials with similar rheological properties, but different compositions and structures, offering a unique opportunity to establish possible correlations between cell activity and substrate properties. For this we used NHDF cells that were seeded and grown for 72 ​h. Their metabolic activity was evaluated by the Alamar Blue test. The pure collagen at 20 ​mg ​mL^−1^ after 24 ​h of contact with NHDFs was used as the 100% reference.

**Fig. 5:**
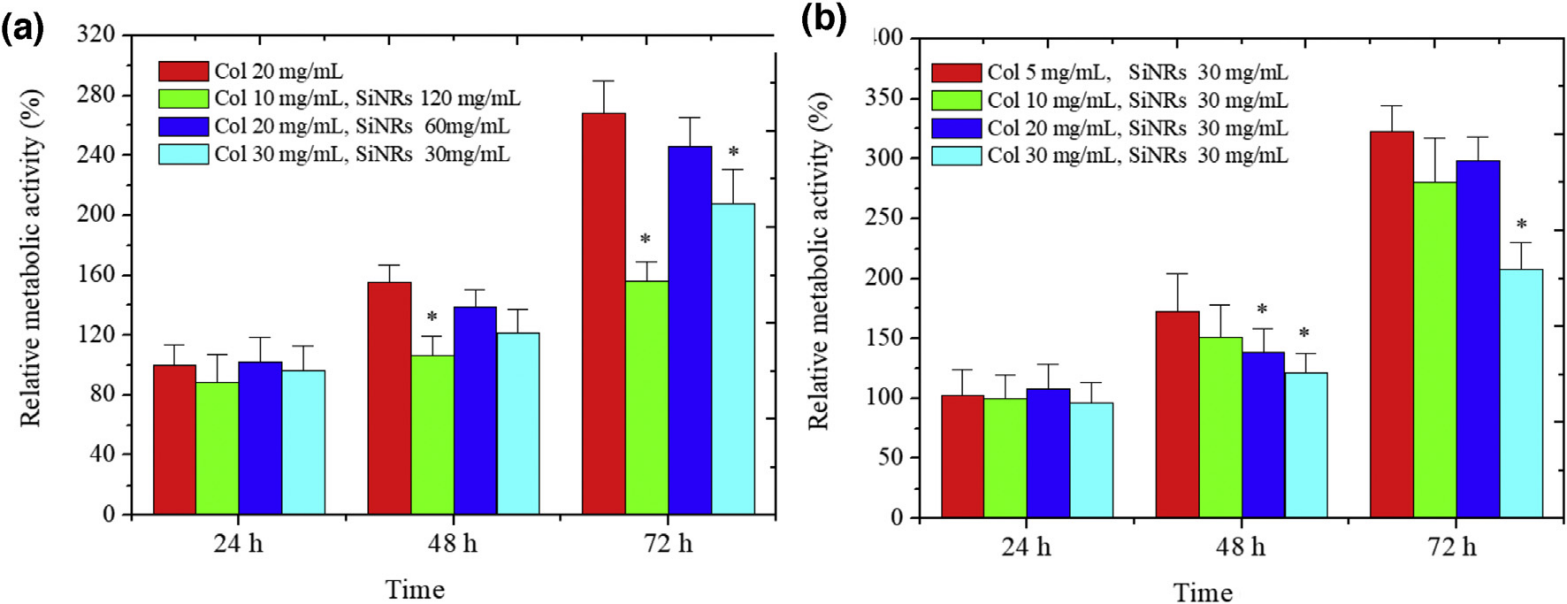
Metabolic activity of surface-cultured fibroblasts (Alamar Blue assay) (a) at constant G′ value and (b) at constant SiNRs content. Control collagen hydrogels (20 ​mg.mL-1) at day 1 were normalized to 100%. Variance of the relative metabolic activity between the hydrogels at 24 ​h, 48 ​h, and 72 ​h was determined by one-way ANOVA with Turkey post hoc test, ^∗^P ​< ​0.05. SiNRs, silica nanorods; ANOVA, analysis of variance.

First, when comparing materials exhibiting similar *G’* values (*ca*. 2 ​kPa), no significant difference was observed after 24 ​h, suggesting that cell adhesion was similar in all cases (Fig. 5a). After 48 ​h, cell proliferation has occurred for all samples to a similar extent, except for sample with low collagen (10 ​mg ​mL^−1^) and high SiNR (120 ​mg ​mL^−1^) content. This difference was even more marked after 72 ​h, but sample with high protein (30 ​mg ​mL^−1^) and low silica (30 ​mg ​mL^−1^) contents also showed lower cell activity. The low activity measured for the highest SiNRs concentration may have originated from some cytotoxic effect of silica rods, but we also noticed a decrease in activity for the sample with the lower silica content while intermediate silica content does not induce any detrimental effect. Therefore the possible cytotoxicity of SiNRs is not the main parameter to consider to explain these results.

Aiming at better understanding of these results, we compared a series of composite prepared at constant SiNR concentration (30 ​mg ​mL^−1^) but various collagen contents. As shown in Fig. 5b, the initial cell adhesion is similar for all hydrogels. After 48 ​h, cell proliferation has occurred but to an extent that decreases with increasing collagen concentration. After 72 ​h, the cellular activity is the same for all samples except for the 30 ​mg ​mL^−1^ collagen composite. Again, if this detrimental effect was only related to silica, then we could have expected that it would be more marked for the highest silica:collagen ratio. In fact, we observe the opposite trend.

**Fig. 6:**
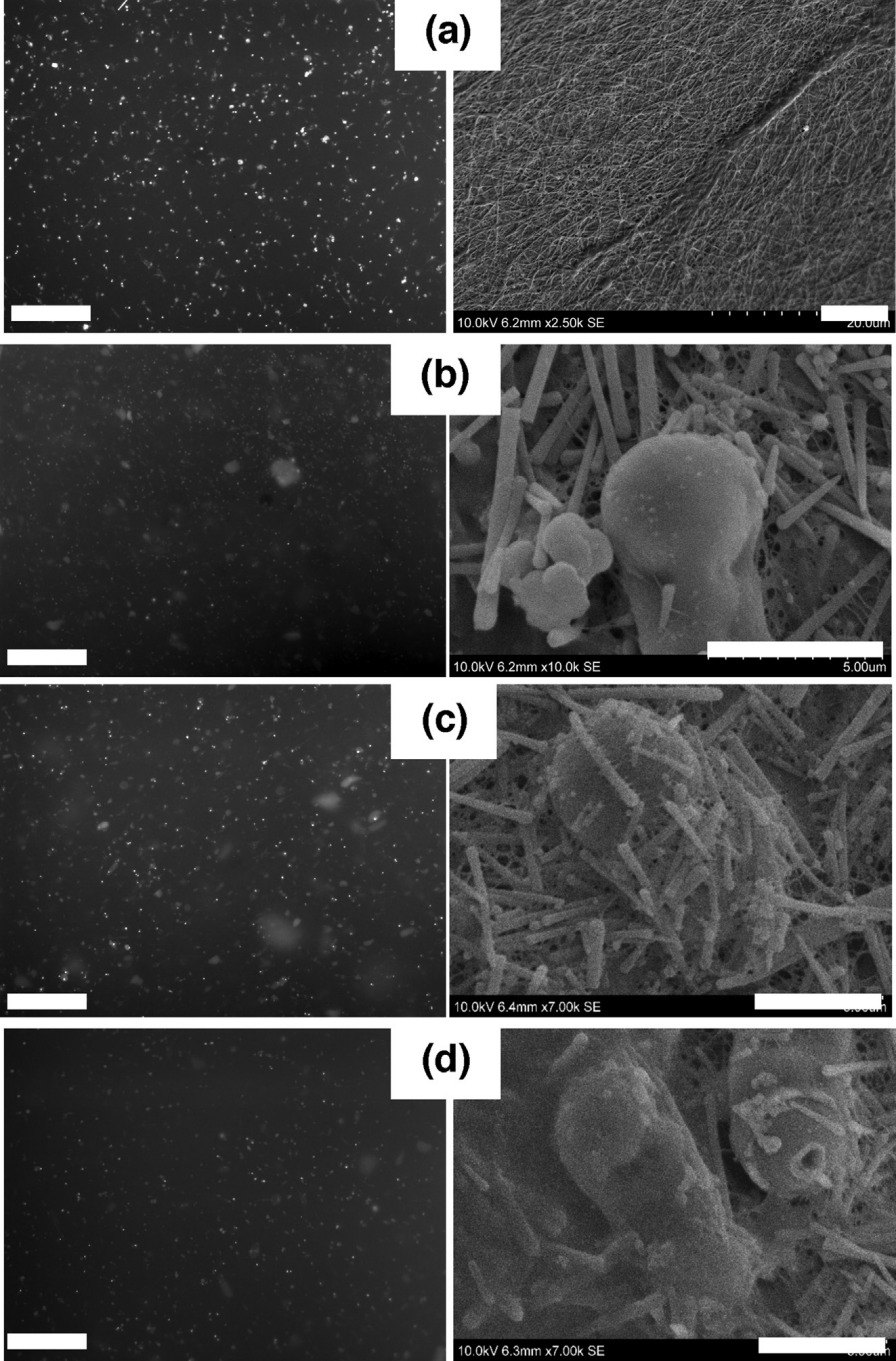
Fluorescence (left-hand column; scale bar ​= ​600 ​μm) and SEM (right-hand column; scale bar ​= ​5 ​μm) images of NHDFs incubated for 72 ​h with (a) 20 ​mg ​mL^−1^ collagen, (b) 10 ​mg ​mL^−1^ collagen, 120 ​mg ​mL^−1^ SiNRs, (c) 20 ​mg ​mL^−1^ collagen, 60 ​mg ​mL^−1^ SiNRs, and (d) 30 ​mg ​mL^−1^ collagen, 30 ​mg ​mL^−1^ SiNRs. For fluorescence imaging, cell nuclei were stained with DAPI. Because of high background due to SiNR, images are presented in grey scale. NHDFs, normal human dermal fibroblasts; SEM, scanning electron microscopy; SiNRs, silica nanorods; DAPI, 4,6-diamidino-2-phenylindole.

We then used fluorescence microscopy and SEM to image the different samples. Selected images for the first series of samples with similar *G’* but different compositions after 72 ​h (data after 24 ​h are provided as Supplementary Fig. S4) were shown in Fig. 6. For fluorescence, cell nuclei were stained with DAPI. However, because of high background due to SiNRs (see Supplemental Fig. S5), images are presented in grey scale.

For the pure collagen, the extent of proliferation between 24 ​h and 72 ​h is validated by fluorescence microscopy. It is also quite clear that all cells are not in the same focus plan, especially after 72 ​h, suggesting that colonization of the matrix has started. In the case of the composites, the presence of the silica rods made the imaging more difficult. However, it is possible to confirm that very few cells are present on the low collagen/high silica sample and even less are observed on high collagen/low silica compared to intermediate composition after 72 ​h.

SEM imaging provided complementary information. The first observation is that silica rods are found in very large amount at the surface of the composite, in contrast to the initial cell-free SEM images shown in Fig. 2. A second interesting point is seen for the composite with intermediate collagen and silica content where cells appear coated by a mixture of collagen fibrils and rods. It seems that cells have strongly interacted and remodeled the composite hydrogel surface and/or penetrated the network. However, NHDFs grown on the composite surfaces have a round shape which indicates poor adhesion, suggesting that silica particles have a detrimental effect on their adhesion.

**Fig. 7:**
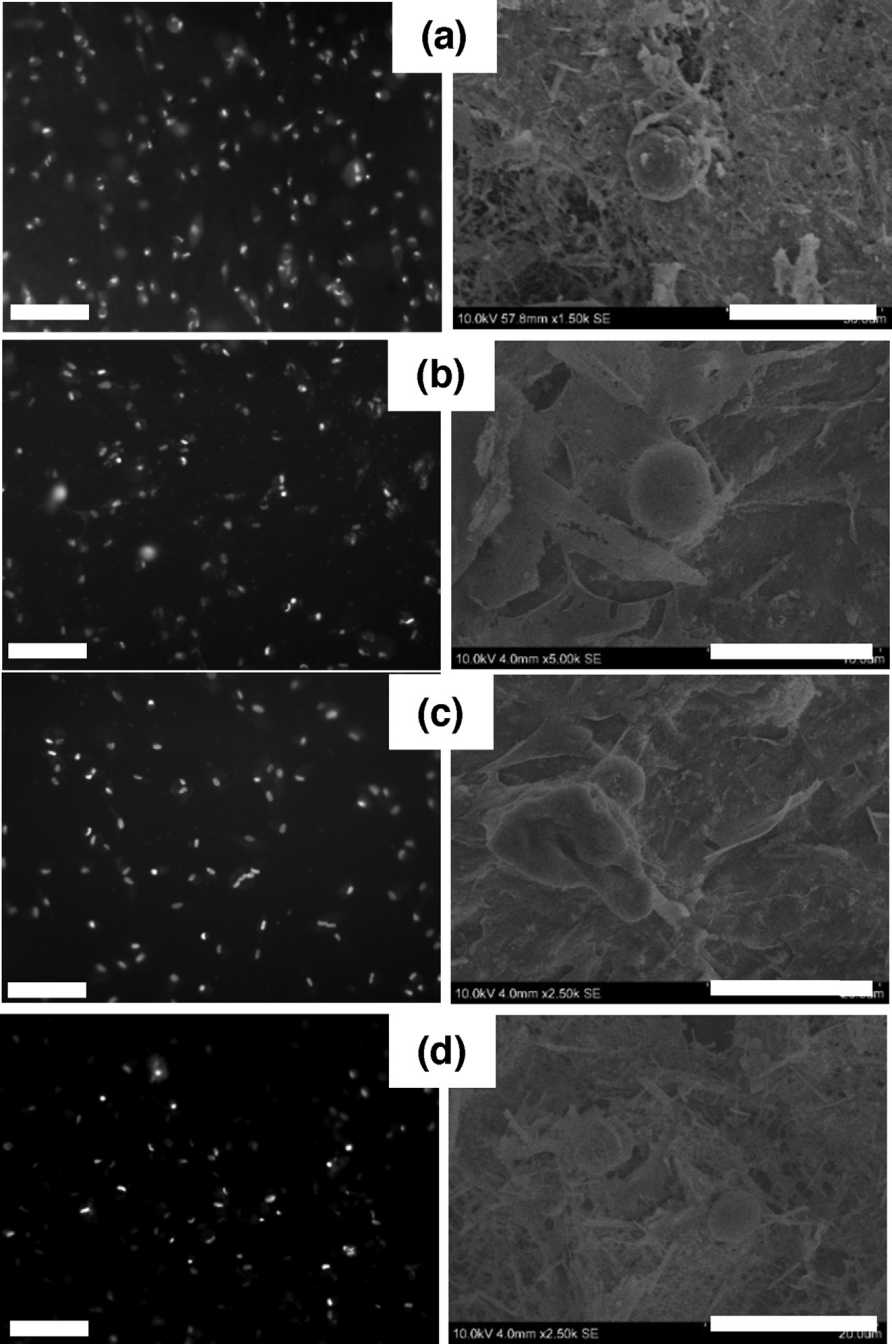
Fluorescence (left-hand column; scale bar ​= ​300 ​μm) and SEM (right-hand column; scale bar ​= ​20 ​μm) images of NHDFs incubated for 72 ​h with (a) 5 ​mg/mL collagen, (b) 10 ​mg/mL collagen, (c) 20 ​mg/mL collagen, and (d) 30 ​mg/mL collagen, all supplemented with 30 ​mg ​mL^−1^ SiNRs. For fluorescence imaging, cell nuclei were stained with DAPI. Because of high background due to SiNR, images are presented in grey scale. SEM, scanning electron microscopy; SiNRs, silica nanorods; DAPI, 4,6-diamidino-2-phenylindole; NHDFs, normal human dermal fibroblasts.

The key role played by the collagen matrix itself was evidenced in the second series of composite hydrogels using a constant SiNR concentration of 30 ​mg ​mL^−1^ (Fig. 7). Fluorescent imaging allows pointing out the decrease in cell density with increasing collagen concentration after 72 ​h. Thanks to the low SiNR content, it is also possible to better observe cells that have penetrated inside the matrix. In SEM images, highly adhering well-spread NHDFs are observed after 72 ​h for all composites except for 30 ​mg ​mL^−1^ collagen, in agreement with cellular activity measurements. These results indicate that, independently of the silica amount, high collagen concentration can also be detrimental to cell viability.

## 4 Discussion

### 4.1 The binary collagen–SiNR interface

Associating silica (nano)rods with type I collagen at different absolute and relative concentrations allows the preparation of nanocomposite hydrogels with very different structures and rheological behaviors. It is important to consider that the *G’* value reflects the response of the collagen or composite network to a shearing force. In pure collagen, the network cohesion is insured by interfibrillar interactions, and its strength increases with fibril density [5]. When rods are added, several effects can occur: (i) rods can perturb the fibrillar organization of collagen and weaken its cohesion, (ii) rods can bridge the fibrils and increase the cohesion of the composite structure, and (iii) interactions between rods can contribute, either positively or negatively, to the response of the composite network: while small aggregates may constitute weak points in the structure, larger ones may, on the contrary, resist the shearing force.

One important and surprising information obtained from TEM images is the ability to the rods to orient the collagen fibers. To our knowledge, such an orientation was never reported before for collagen self-assembly in the presence of anisotropic particles. This supports the existence of strong interactions between the protein and the rods ​that are expected to have a major role in the rheological properties of the composite.

**Fig. 8:**
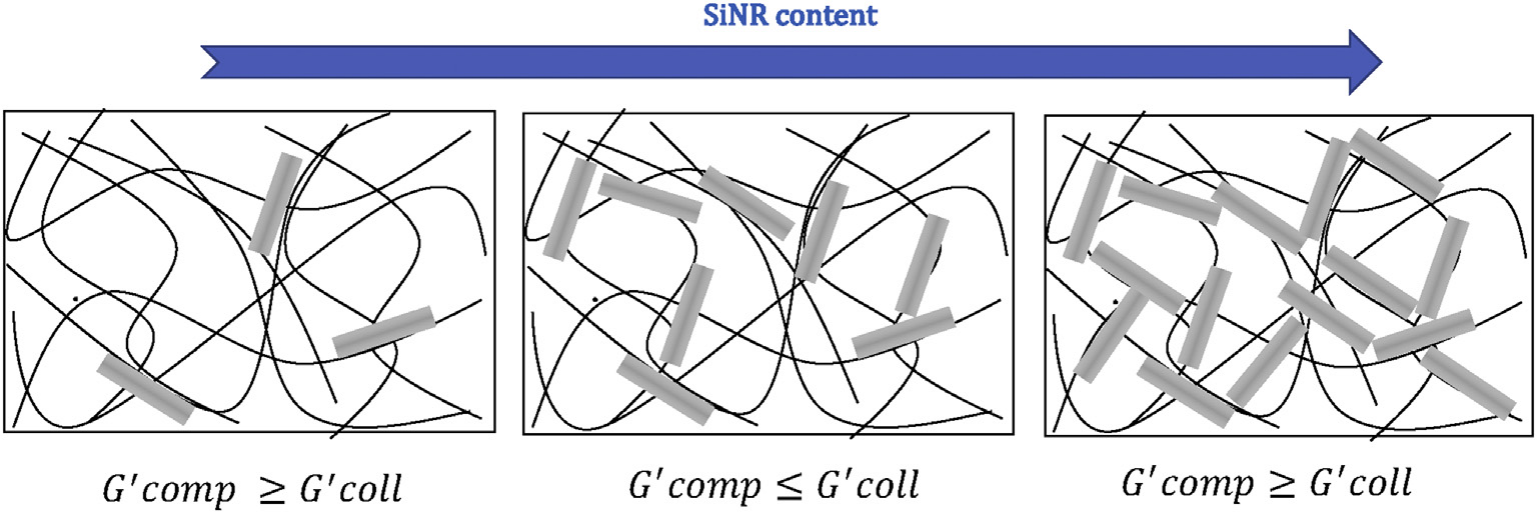
Schematic evolution of elastic modulus of composite hydrogels with nanorods concentration at fixed collagen content. SiNR, silica nanorod.

The inverted bell shape of the variations of G′ with SiNRs concentration at fixed collagen concentration illustrates the balance of interactions at stake. Introduction of silica rods in low amounts in the collagen network may destabilize the interfibril interactions, but this can be at least partially compensated by favorable local silica–collagen interactions. If silica amount increases, SiNR–SiNR unfavorable interactions become prevalent and weaken the composite network. However, at higher silica concentration, the network mainly consists of close-packed silica rods that can be favorably bridged by collagen. (Fig. 8). The fact that the SiNR concentration for minimal *G’* decreases with increasing collagen concentration then reflect that denser protein networks are more efficient in stabilizing rods aggregates, which explains why little variations were observed for the low concentrated 5 ​mg ​mL^−1^ hydrogels. These results show that strong charge/matrix interactions are involved in the stiffening of the materials, highlighting their true composite nature.

So far, collagen–silica nanocomposites prepared through a similar method were only described for protein concentration of 5 ​mg ​mL^−1^ or below and spherical nanoparticles at a maximum amount of 60 ​mg ​mL^−1^ so that direct comparison with this new set of data is not straightforward [34], [37], [39]. However, these data pointed out that the smallest nanoparticles (*ca*. 12 ​nm) were the most effective in enhancing the *G’* value of the collagen network, whereas larger ones (between 80 ​nm and 450 ​nm) had no significant effect. This was attributed to the larger surface area of the smallest colloids, favoring their interaction with collagen. Here-studied rods have a volume equivalent to spheres of *ca*. 300 ​nm in radius, a size for which such extended interaction would not be expected. However, it appears that their rod-like morphology, that allows collagen fibril formation at their surface, is responsible for their observed noticeable impact on the rheological properties of the composite structure.

### 4.2 The ternary NHDF–collagen–silica interface

Focusing now on NHDF behavior, our results show that materials with similar *G’* value impact differently on their adhesion and proliferation, in a way that is not directly proportional to the amount of silica particles. Furthermore, keeping the SiNR constant, high collagen concentrations are detrimental to cellular activity.

**Fig. 9:**
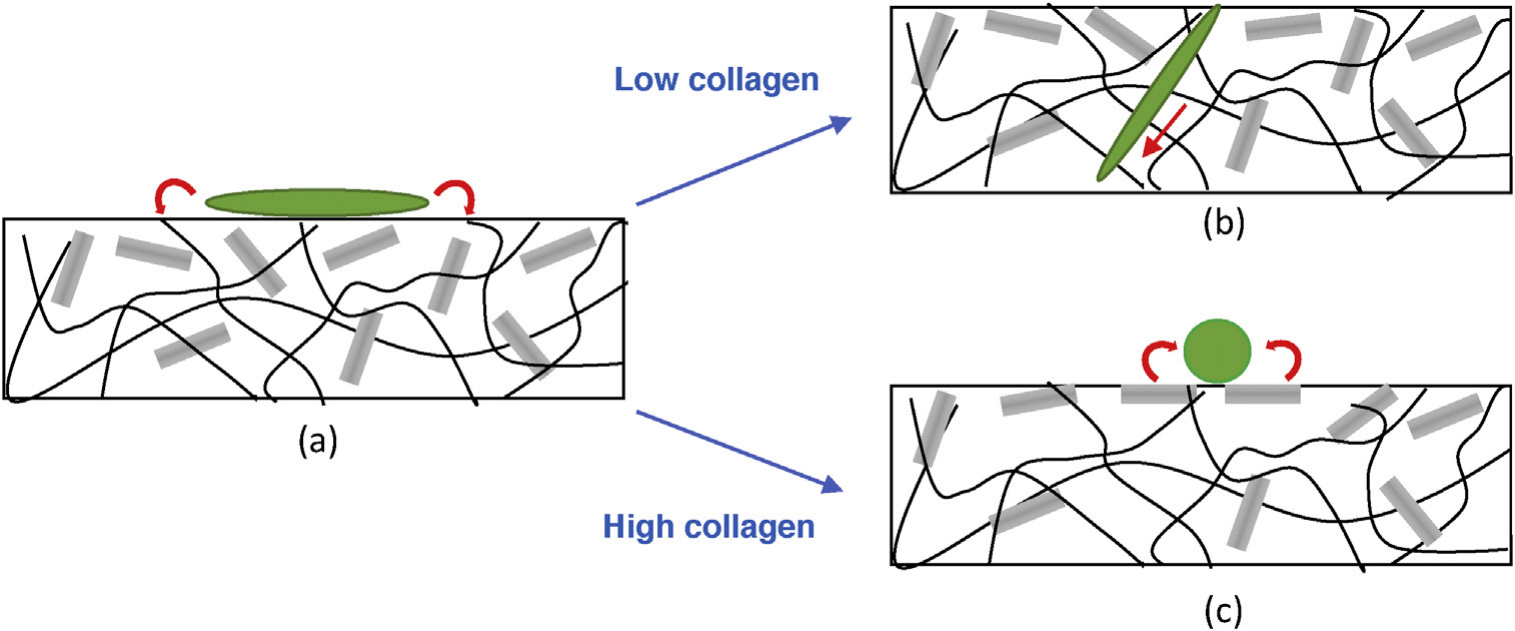
Schematic fate of fibroblasts seeded on nanocomposites: (a) adhesion is favored by interaction with surface collagen; (b) at low collagen content, cells can penetrate the hydrogel; and (c) at high collagen concentration, cells cannot proliferate because of unfavorable interactions with nanorods.

First, such an inhibition of NHDF proliferation in the presence of collagen–silica nanocomposites was not reported previously. As pointed out previously, direct comparison with the literature is difficult because reported protein and particle concentration do not overlap with the present ones. However, it is again possible to assume that the shape of the silica particles has a strong influence on their interaction with NHDFs since the length of the rods (*ca*. 3 ​μm) is in the same order of magnitude as fibroblasts dimensions (10–15 ​μm). Concerning the effect of collagen concentration, the most straightforward explanation is related to its previously reported influence on hydrogel colonization. It has been shown that the rate of penetration of NHDFs within collagen hydrogels, that involves their degradation by metalloprotease enzymes, decreases with increasing protein concentration [46]. Altogether, our data suggest that cell adhesion and proliferation is not favored on the composite surface where silica rods are found in large amounts. In composites with low collagen concentration, NHDFs may be able to penetrate the protein network and find a favorable environment for proliferation. In contrast, at high collagen content, fibroblasts are stuck on the surface and their proliferation is hindered by the presence of silica rods (Fig. 9).

Considering the ternary cell–protein–mineral interface, a final point to discuss is whether the collagen–silica interactions have *per se* a direct effect on NHDF behavior, beyond their impact on the rheological properties of the materials. We have pointed out earlier a difference in SEM images of the nanocomposites before and after cell seeding. Nanorods that were initially sometimes difficult to distinguish within the collagen network became clearly visible on the surface after cell adhesion. Considering that the protein phase is the most favorable for cell adhesion, that is usually followed by a stage of contraction/remodeling [49], it can be hypothesized that collagen fibrils initially adsorbed on the nanorods surface are detached by the action of cells, leaving bare silica surface. This would in turn indicate that, whereas silica–collagen interactions are strong enough to contribute to the composite resistance under the shearing stress of the rheological measurements, they cannot sustain the pulling force exerted by the adhering fibroblasts.

## 5 Conclusions

By combining, for the first time, silica (nano)rods with type I collagen, it was possible to obtain nanocomposite hydrogels with variable structures. Rheological studies and EM imaging evidenced that strong interactions exist between silica and collagen, enlightening that these materials can be truly considered as composite structures at the macroscopic scale. However, NHDF cells do discriminate between the protein and the mineral phases, with no clear impact of the collagen–silica interactions. In other words, the cells' response to nanocomposite environments cannot be extrapolated only from global structural and mechanical characteristics, owing to their ability to interact in a specific manner with each of the component. This can have a large impact on the *in vivo* fate of such nanocomposite biomaterials, in particular considering their colonization/biodegradation rates. Furthermore, whereas, in the present system, silica appears not to have no direct biological influence on fibroblasts, the picture should become more complex when bioactive particles are used as the mineral phase of the nanocomposite material.

## Funding

This work was supported by a PhD grant from the China Scholarship Council.

## Conflict of interest

The authors declare that they have no known competing financial interests or personal relationships that could have appeared to influence the work reported in this paper.

## Data availability

The raw/processed data required to reproduce these findings cannot be shared at this time because of technical or time limitations.
