# Supplementary figures and images for: Exploring the cell–protein–mineral interfaces: Interplay of silica (nano)rods@collagen biocomposites with human dermal fibroblasts
Source: Mater Today Bio. 2019 Apr 8;1:100004. doi: 10.1016/j.mtbio.2019.100004 (PMC7061546; doi:10.1016/j.mtbio.2019.100004)

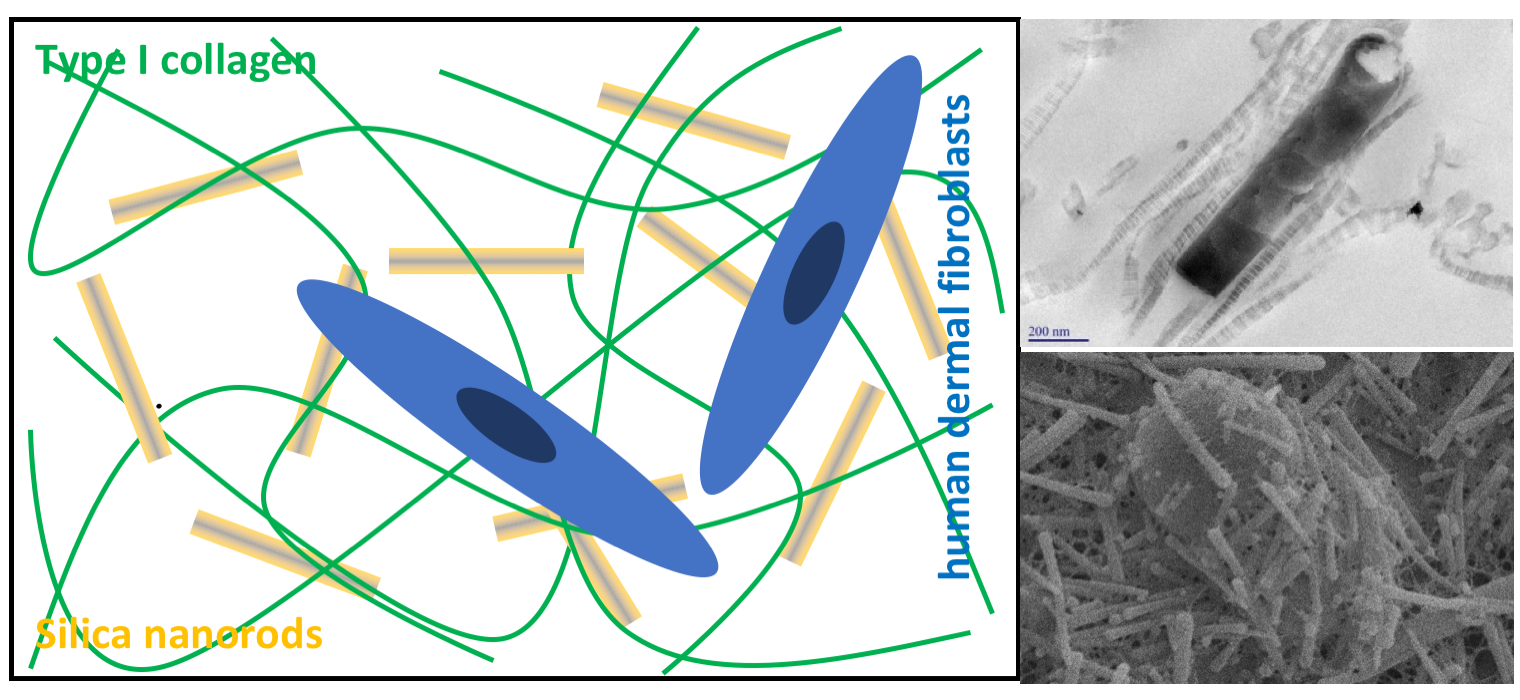

Supplement: Multimedia component 2 [file mmc2.zip › mtbio_100004_MatTodBio-YS-GrAbs_mmc2.tiff]
